# Polymorphisms Influencing Expression of Dermonecrotic Toxin in *Bordetella bronchiseptica*

**DOI:** 10.1371/journal.pone.0116604

**Published:** 2015-02-02

**Authors:** Keisuke Okada, Hiroyuki Abe, Fumio Ike, Yoshitoshi Ogura, Tetsuya Hayashi, Aya Fukui-Miyazaki, Keiji Nakamura, Naoaki Shinzawa, Yasuhiko Horiguchi

**Affiliations:** 1 Department of Molecular Bacteriology, Research Institute for Microbial Diseases, Osaka University, Osaka, Japan; 2 Experimental Animal Division, RIKEN BioResource Center, Ibaraki, Japan; 3 Division of Microbial Genomics, Department of Genomics and Bioenvironmental Science, Frontier Science Research Center, University of Miyazaki, Miyazaki, Japan; 4 Division of Microbiology, Department of Infectious Diseases, Faculty of Medicine, University of Miyazaki, Miyazaki, Japan; Universidad Nacional de La Plata., ARGENTINA

## Abstract

*Bordetella bronchiseptica* is a pathogenic bacterium causing respiratory infections in a broad range of mammals. Recently, we determined the whole genome sequence of *B. bronchiseptica* S798 strain isolated from a pig infected with atrophic rhinitis and found four single-nucleotide polymorphisms (SNPs) at positions -129, -72, +22, and +38 in the region upstream of *dnt* encoding dermonecrotic toxin (DNT), when compared with a rabbit isolate, RB50. DNT is known to be involved in turbinate atrophy observed in atrophic rhinitis. Immunoblotting, quantitative real-time PCR, and β-galactosidase reporter assay revealed that these SNPs resulted in the increased promoter activity of *dnt* and conferred the increased ability to produce DNT on the bacteria. Similar or identical SNPs were also found in other pig isolates kept in our laboratory, all of which produce a larger amount of DNT than RB50. Our analysis revealed that substitution of at least two of the four bases, at positions -72 and +22, influenced the promoter activity for *dnt*. These results imply that these SNPs are involved in the pathogenicity of bordetellae specific to pig diseases.

## Introduction

Pathogenic bordetellae including *Bordetella bronchiseptica*, *B*. *pertussis*, and *B*. *parapertussis* are known to cause respiratory infections in mammals. These bordetellae share many virulence factors, such as filamentous hemagglutinin, adenylate cyclase toxin, dermonecrotic toxin (DNT), and type III secretion apparatus and effectors. Comparative analysis of their genome sequences revealed that *B*. *pertussis* and *B*. *parapertussis* independently evolved from a *B*. *bronchiseptica*-like ancestor [1] through genome decay including extensive loss and translocation of genes, which resulted in different host tropism: *B*. *bronchiseptica* infects various mammals, causing atrophic rhinitis in pigs, kennel cough in dogs, bronchopneumonia in guinea pigs and rabbits, and sporadic infections in humans. *B*. *pertussis* and *B*. *parapertussis* of one lineage infect humans exclusively, while *B*. *parapertussis* of another distinct lineage is found only in sheep [2]. These bordetellae, which are phylogenetically closely related to each other but show different host tropism, are considered to serve as an appropriate subject of research for understanding how host tropism of pathogenic bacteria could be determined [1,3–5].

Recently, we determined the whole genome sequence of *B*. *bronchiseptica* S798 strain isolated from a pig infected with atrophic rhinitis [6], the strain that has often been used for the production and purification of DNT [7,8] and analyses of its pathogenesis [9,10]. This was the first sequenced genome of a *B*. *bronchiseptica* pig isolate. To gain insight into genetic characteristics that may reflect the host tropism of *B*. *bronchiseptica*, we compared the S798 genome with previously sequenced genomes of rabbit, dog, and human isolates, but did not find any S798–specific genetic loci that could be related to the bacterial pathogenesis. Instead, four single-nucleotide polymorphisms (SNPs) were found in the region upstream of *dnt*, when compared with a rabbit isolate, RB50, which has been widely used as a reference strain [1,11]. In this study, we examined the effects of the SNPs on bacterial phenotype and found that they resulted in the increased promoter activity of *dnt*, leading to the high production of DNT, which is known to cause turbinate atrophy, by which atrophic rhinitis is characterized [8]. Further analyses revealed that 12 of 13 pig isolates carried 3 to 4 SNPs at the same sites and produced larger amounts of DNT than the other strains of *B*. *bronchiseptica* and *B*. *pertussis* having no nucleotide substitutions at the corresponding positions. These results might imply a correlation between genetic characteristics and pathogenesis to pigs in *B*. *bronchiseptica*.

## Materials and Methods

### Bacterial strains and growth media

The bacterial strains used in this study are listed in S1 Table. *B*. *bronchiseptica* pig isolates S798, R033, S807, AFUY13, KII13, KII24, KG19, KHI11, MH110, TYA12, TYA13, STY136, and STY137, which were field isolates from pigs, were provided by N. Terakado (National Institute of Animal Health, Ibaraki, Japan) and S. Nagai (Nippon Institute for Biological Science, Tokyo, Japan). Four strains of *B*. *bronchiseptica* 10177–36, 10255–6, 10519–15, and 10092–26 were isolated from mice in Japan. *B*. *bronchiseptica* RB50 was provided by P. A. Cotter (University of North Carolina, NC). *B*. *pertussis* Tohama was maintained in the laboratory. The *Bordetella* strains were grown at 37°C on a Bordet-Gengou (BG) agar plate (Becton Dickinson, Franklin Lakes, NJ) containing 0.8% (v/v) glycerol and 20% (v/v) defibrinated horse blood or in Stainer-Scholte (SS) liquid medium [12]. *Escherichia coli* strains were grown at 37°C on an LB agar plate or LB broth. The growth media were supplemented with antibiotics at the following concentrations when necessary: gentamicin, 10 μg/ml; ceftibuten, 10 μg/ml; streptomycin, 10 μg/ml; and ampicillin, 50 μg/ml. The concentration of bacterial cells was estimated from OD_650_ values according to the following equation: 1 OD_650_ = 4.5 x 10^8^ cells/ml.

Mutant strains of *B*. *bronchiseptica* were constructed as described previously by Sekiya *et al*. [13] with slight modifications. The plasmids and primers used in this study are listed in S2 and S3 Tables. A single-base substitution was introduced into the region upstream of *dnt* as follows. A 0.5-kbp DNA fragment to generate the single-base substitution was amplified by PCR with *B*. *bronchiseptica* S798 genomic DNA as a template and the combination of primers S798-DNTr-Up-S and S798-DNTr-Down2-AS. The single-base substitution was generated by the underlined base of the primer (S3 Table). A 0.7-kbp DNA fragment was amplified by PCR with *B*. *bronchiseptica* S798 genomic DNA as a template and the combination of primers S798-DNTr-Up2-S and S798-DNTr-Down-AS. Both DNA fragments were joined by PCR with the combination of primers S798-DNTr-Up-S+ and S798-DNTr-Down-AS+. The resulting PCR product contained sequences corresponding to those of the terminal regions of *Sma*I-digested pABB-CRS2-GmA2, which was used as a suicide vector at each end. The PCR product was ligated with pABB-CRS2-GmA2 via the overlapping sequences using the In-Fusion HD cloning kit (Takara Biochemicals, Shiga, Japan) according to the manufacturer’s instructions. The resulting plasmid was designated pABB-CRS2-P*dnt*-72C and introduced into *E*. *coli* DH5α λ*pir*, and then transferred to *B*. *bronchiseptica* RB50 by triparental conjugation with a helper strain, HB101/pRK2013. The first recombinant strain that had integrated pABB-CRS2-P*dnt*-72C into the chromosome was selected by growth on BG agar plates containing ceftibuten and gentamicin. Subsequently, the second recombinant strains, in which pABB-CRS2-P*dnt*-72C was eliminated by homologous recombination, were isolated, after incubation on LB agar plates without NaCl and containing 10% sucrose, ceftibuten, and streptomycin. PCR with the combination of the genomic DNA of recombinant strains as templates and primers *Pst*I-GmR-S and *Pst*I-GmR-AS did not amplify any DNA fragments, confirming elimination of pABB-CRS2-P*dnt*-72C.

### DNA sequencing

Genomic DNA was isolated using the Genomic DNA Buffer Set and Genomic-tip 100/G (Qiagen, Venlo, Netherlands). A DNA fragment containing *dnt* and its flanking regions was amplified by PCR with *B*. *bronchiseptica* genomic DNA as a template and the combination of primers BB3979-S and BB3975-AS. The resulting PCR product was isolated by agarose gel electrophoresis, purified using a QIAquick Gel Extraction Kit (Qiagen), and sequenced with the primers BB3979-AS_reverse and BB3979-S-2. Multilocus Sequence Typing (MLST) analysis on the basis of the sequence of seven housekeeping genes (*adk*, *fumC*, *glyA*, *tyrB*, *icd*, *pepA*, and *pgm*) was performed as previously described [4]. Sequence types of each strain were designated according to the *Bordetella* MLST database (http://pubmlst.org/bordetella).

### Immunoblotting

*B*. *bronchiseptica* and *B*. *pertussis* grown in SS medium for 12 h and 24 h, respectively, were recovered by centrifugation at 12,000 rpm for 5 min, suspended in sodium dodecyl sulfate (SDS) gel-loading buffer (62.5 mM Tris-HCl, pH6.8, containing 10% glycerol, 2% SDS, 25 mM dithiothreitol, and 0.005% bromophenol blue), sonicated, and subjected to SDS polyacrylamide gel electrophoresis (7.5% polyacrylamide) [14] after incubation at 98°C for 5 min, followed by electrotransfer to a polyvinylidene difluoride membrane (Millipore Billerica, MA). The membrane was probed with the combination of mouse monoclonal antibody against DNT [15] and horseradish peroxidase-conjugated goat anti-mouse IgG antibody (Cappel, MP Biomedicals, Santa Ana, CA), or another combination of rabbit antiserum against FtsZ, a bacterial cytoskeletal protein, and horseradish peroxidase-conjugated goat anti-rabbit IgG antibody (Jackson ImmunoResearch Laboratories, Inc., West Grove, PA). The proteins on the membrane were detected with an ECL Western Blotting Detection System (GE Healthcare, Uppsala, Sweden). DNT purified from *B*. *bronchiseptica* S798 as described previously [16] was used as a positive control.

### Quantitative RT-PCR

Bacterial RNA was isolated with TRIzol (Ambion, Life Technologies) according to the manufacturer’s instructions. Contaminating genomic DNA was digested by RNase-free DNase I (Takara). RNA was reverse-transcribed with SuperScript III Reverse Transcriptase (Invitrogen, Life Technologies, Carlsbad, CA) with random primers. With the resulting cDNA as a template, *dnt* and *recA* were amplified using Power SYBR Green PCR Master Mix (Applied Biosystems, CA) with primer pairs RT-3978-F/RT-3978-R and RT-*recA*-F/RT-*recA*-R, respectively. The relative levels of *dnt* mRNA in each strain were normalized to the corresponding *recA* levels by the comparative cycle threshold method [17].

### 5’-Rapid amplification of cDNA ends (RACE)

The transcriptional start site of *dnt* was determined by sequencing of the 5’ terminal region of mRNA, which was cloned using the 5’-Full RACE Core set (Takara) according to the manufacturer’s instructions. A DNA fragment complementary to the 5’ terminal region of *dnt* mRNA was obtained by reverse transcription with 5’ end-phosphorylated primer DNT-5RACE-RT and circularized by T4 RNA ligase. A 0.3-kbp DNA fragment containing the sequence corresponding to the 5’ terminal region of *dnt* mRNA was amplified by nested PCR with the circularized complementary DNA as a template and two combinations of the primers: DNT-5RACE-S1 and DNT-5RACE-A1, or DNT-5RACE-S2 and DNT-5RACE-A2. The resulting PCR product was cloned into pCR2.1-TOPO (Invitrogen) and sequenced.

### β-galactosidase reporter assay

A reporter plasmid, pT1T2-SD-*lacZ*, was constructed and used for a β-galactosidase reporter assay (S1 Fig.). A 3.8-kbp DNA fragment containing genes required for plasmid replication (*rep*), resistance to gentamicin (GmR), and plasmid mobilization (*mob*) and an SD sequence, which is a ribosome-binding site in the mRNA, was amplified by inverse PCR with pBBR1MCS-5 as a template and the combination of primers *Xho*I-pBBR1MCS-AS+ and SD-*Sma*I-pBBR1MCS-S+. A 0.4-kbp DNA fragment containing the BBr01 promoter, for 16S ribosomal RNA, was amplified by PCR with *B*. *bronchiseptica* RB50 genomic DNA as a template and the combination of primers *Xho*I-PBBr01-SD-S+ and *Sma*I-PBBr01-SD-AS+. The DNA fragment contained sequences corresponding to those of the terminal regions of the fragment of the portion of pBBR1MCS-5 at each end. These corresponding sequences were generated from the underlined 15-base sequences of the primers (S3 Table). Both DNA fragments were ligated via the overlapping sequences using the In-Fusion HD cloning kit (Takara), and pBBr01-SD was obtained. A 3.1-kbp DNA fragment containing *lacZ* was amplified by PCR with *E*. *coli* W3110 genomic DNA as a template and the combination of primers *Ecoli*-*lacZ*-S+ and *Ecoli*-*lacZ*-AS+. The resulting PCR product was cloned into *Sma*I-digested pBBr01-SD using the In-Fusion HD cloning kit (Takara), and pBBr01-SD-*lacZ* was obtained. A 7.3-kbp DNA fragment was amplified by inverse PCR with pBBr01-SD-*lacZ* as a template and the combination of primers Promoter-T1-S and Promoter-T1-AS, and linearized pBBr01-SD-*lacZ* was obtained. A 0.3-kbp DNA fragment containing *rrnB* terminator was amplified by PCR with pKK232–8 (Amersham, GE Healthcare, Uppsala, Sweden) as a template and the combination of primers *rrnB*-T1-termi-S2+ and *rrnB*-T2-termi-AS2+. The resulting PCR product was joined with linearized pBBr01-SD-*lacZ* using the In-Fusion HD cloning kit (Takara), and pT1T2-BBr01-SD-*lacZ* was obtained. A 7.3-kbp DNA fragment without a BBr01 promoter was amplified by inverse PCR with pT1T2-BBr01-SD-*lacZ* as a template and the combination of primers SD-*lacZ*-S and SD-*lacZ*-AS, and linearized pT1T2-BBr01-SD-*lacZ* without a BBr01 promoter was obtained. The resulting PCR product was phosphorylated by T4 polynucleotide kinase (Takara) and circularized by Ligation high Ver.2 (TOYOBO, Osaka, Japan), and pT1T2-SD-*lacZ* was obtained. For the construction of the *lacZ* fusion with the *dnt* promoter, a 0.2-kbp DNA fragment containing the sequence upstream of *dnt* was amplified by PCR with *B*. *bronchiseptica* genomic DNA as a template and two combinations of primers: DNT-P-SD-S+ and RB50-DNT-P-SD-AS+, or DNT-P-SD-S+ and S798-DNT-P-SD-AS+. The resulting PCR products were independently cloned into linearized pT1T2-BBr01-SD-*lacZ* without a BBr01 promoter using the In-Fusion HD cloning kit (Takara), and pT1T2-RB50, pT1T2-S798, and pT1T2-AFUY13 were obtained. Single-base substitutions were introduced into the upstream region of *dnt* following a standard site-directed mutagenesis protocol (QuickChange, Stratagene, Agilent Technologies, Inc., Santa Clara, CA) with pT1T2-RB50 as a template and four combinations of primers: RB50-DNT-P-72T-S and RB50-DNT-P-72T-AS, RB50-DNT-P-129C-S and RB50-DNT-P-129C-AS, RB50-DNT-P+22T-S and RB50-DNT-P+22T-AS, or RB50-DNT-P+38T-S and RB50-DNT-P+38T-AS, and nine plasmids, pT1T2-RB50–1 to pT1T2-RB50–9, were obtained. Single-base substitutions were generated from the underlined one-base sequence of the primers (S3 Table). *B*. *bronchiseptica* RB50 strains carrying the reporter plasmids were grown in SS medium. One hundred microliters of the bacterial culture was added to one milliliter of 60 mM Na_2_HPO_4_, 40 mM NaH_2_PO_4_, 10 mM KCl, 1 mM MgSO_4_, 50 mM β-mercaptoethanol, 0.001% SDS, and 5% chloroform in a test tube and lysed by thorough shaking. β-galactosidase activity in the bacterial lysate was determined as described previously [18] and obtained values were normalized to OD_650_ values representing bacterial numbers of the respective cultures.

## Results

### SNPs influencing the expression of *dnt* in *B*. *bronchiseptica*

**Fig 1:**
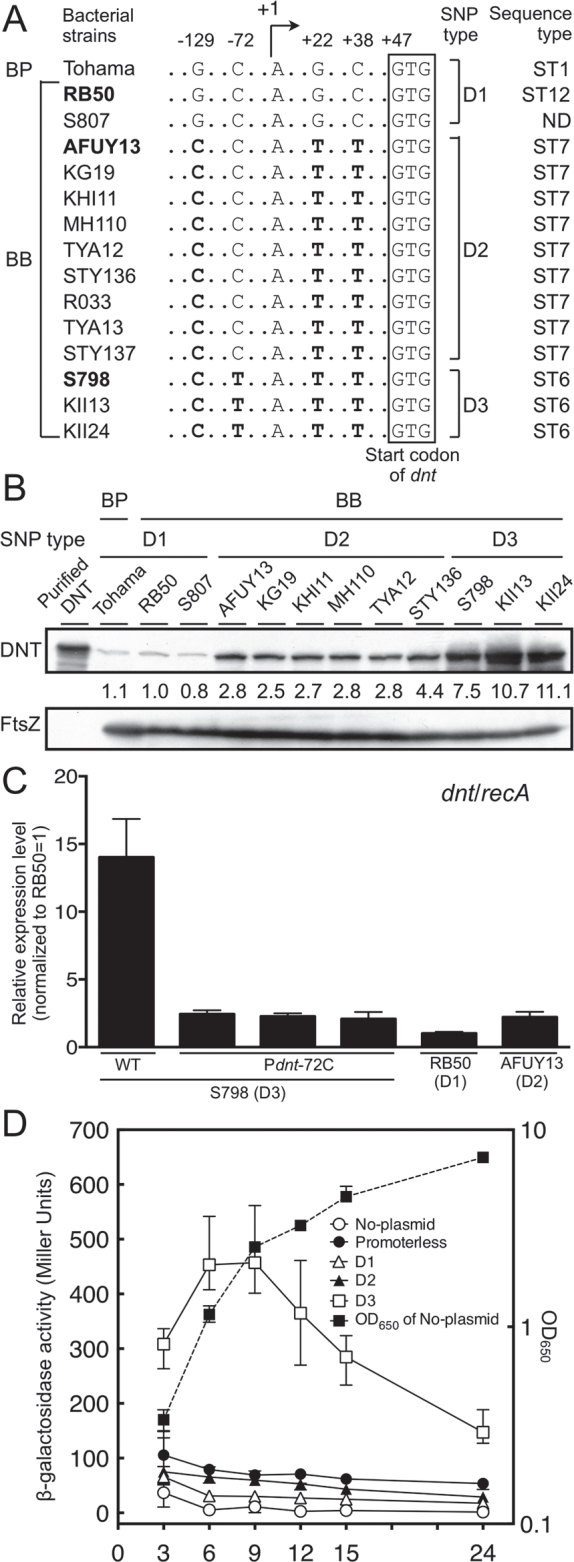
SNPs upstream of *dnt* influence the expression level of DNT. (A) Alignments of SNPs upstream of *dnt*. The positions +1 and +47 indicate the start sites of transcription and translation, respectively. The strains indicated by boldface are hereafter used as representatives for each SNP type. BP and BB indicate *B*. *pertussis* and *B*. *bronchiseptica*, respectively. (B) Detection of DNT in whole-cell lysates by immunoblotting. Whole-cell lysates of indicated strains that were cultivated for 12 h were prepared and subjected to SDS-PAGE, followed by immunoblotting, as described in Materials and Methods. The intensity of each band was measured using Image Gauge Version 4.1 (FUJIFILM Corporation, Tokyo, Japan) and the obtained values of DNT signals were normalized by those of FtsZ signals in each lane, and are expressed below the upper panel as relative intensities when DNT in the sample from RB50 is 1.0. Purified DNT was subjected to SDS-PAGE at 50 ng/lane to indicate the position of the toxin. The results of strains R033, TYA13, and STY137 were omitted but showed similar amounts of DNT as other D2-type strains. (C) Measurement of *dnt* mRNA level by quantitative RT-PCR. Total bacterial RNA was prepared from S798 with D3-type promoter, RB50 with D1-type promoter, AFUY13 with D2-type promoter, and S798P*dnt*-72C, three independent derivatives of S798 having a nucleotide substitution of C for T at position -72 and subjected to quantitative RT-PCR, as described in Materials and Methods. The relative levels of *dnt* mRNA normalized to *recA* mRNA are shown. Each bar represents the mean + standard deviation of three samples. (D) The reporter plasmids harboring a promoter region of D1, D2, or D3 type were introduced into *B*. *bronchiseptica* RB50 and the β-galactosidase reporter assay was carried out after incubation of the bacteria for the indicated periods. Dashed black lines represent growth curves of host strain (No-plasmid) as representative data monitored by OD_650_. Each plot represents the mean ± standard deviation of four samples.

When compared with *B*. *bronchiseptica* RB50, a pig isolate S798 was found to contain four SNPs at positions -129, -72, +22, and +38 in the region upstream of *dnt*, where position +1 is assigned for the transcriptional start site that was determined by the 5’-RACE method (Fig. 1A). Similar SNPs were found in the corresponding regions of the other *B*. *bronchiseptica* strains that were kept in our laboratory. Some strains have the same four SNPs, and the others have three of four at positions -129, +22, and +38. We classified the strains into three groups according to the pattern of SNPs, and designated them as D1 for the strains without the nucleotide substitutions, compared with RB50, D2 for those with the three SNPs, and D3 for the remainder with the four SNPs like S798. S798 strain has been used for purification of DNT because of its high ability to produce the toxin [19]. Therefore, we examined whether the SNPs upstream of *dnt* influence the expression level of the toxin by immunoblotting and quantitative RT-PCR (Fig. 1, B and C). The immunoblotting revealed that the level of DNT produced by the bacteria was increased in the order of D1, D2, and D3 SNP types. The results of the quantitative RT-PCR were consistent with those of the immunoblotting. The level of *dnt* mRNA in S798 (D3 type) was 13.8 times that in RB50 (D1 type) and 6.3 times that in AFUY13 (D2 type). These results indicate that the strains of D3 type have the highest activity of *dnt* transcription, while those of D1 and D2 have the lowest and intermediate activities, respectively. *B*. *pertussis* Tohama strain belonging to D1 type was found to produce as a small amount of DNT as *B*. *bronchiseptica* strains of D1 type (Fig. 1, A and B).

To examine the time course of DNT production, we introduced the reporter plasmids carrying D1-, D2-, and D3-type promoter regions into RB50 strain and carried out the β-galactosidase reporter assay to monitor the transcription activity of each promoter (Fig. 1D). The activity of the D3-type promoter was much higher than those of D1- and D2-type promoters, increased in the early period of incubation to a peak at the mid-log growth phase, and then gradually decreased (Fig. 1D). This time course was in agreement with those described in previous studies that detected DNT activity of cultured *B*. *pertussis* [20] and *B*. *bronchiseptica* [21]. The D1- and D2-type promoter activities were slightly higher than that of the negative control without the reporter plasmids, but did not show any increase over the entire period of incubation. The reporter plasmid without any promoter sequence (promoterless) showed higher activity than those with D1- and D2-type promoters, implying that these promoters might be consistently repressed.

### Base changes at -72 and +22 are crucial for high-level expression of *dnt*

**Fig 2:**
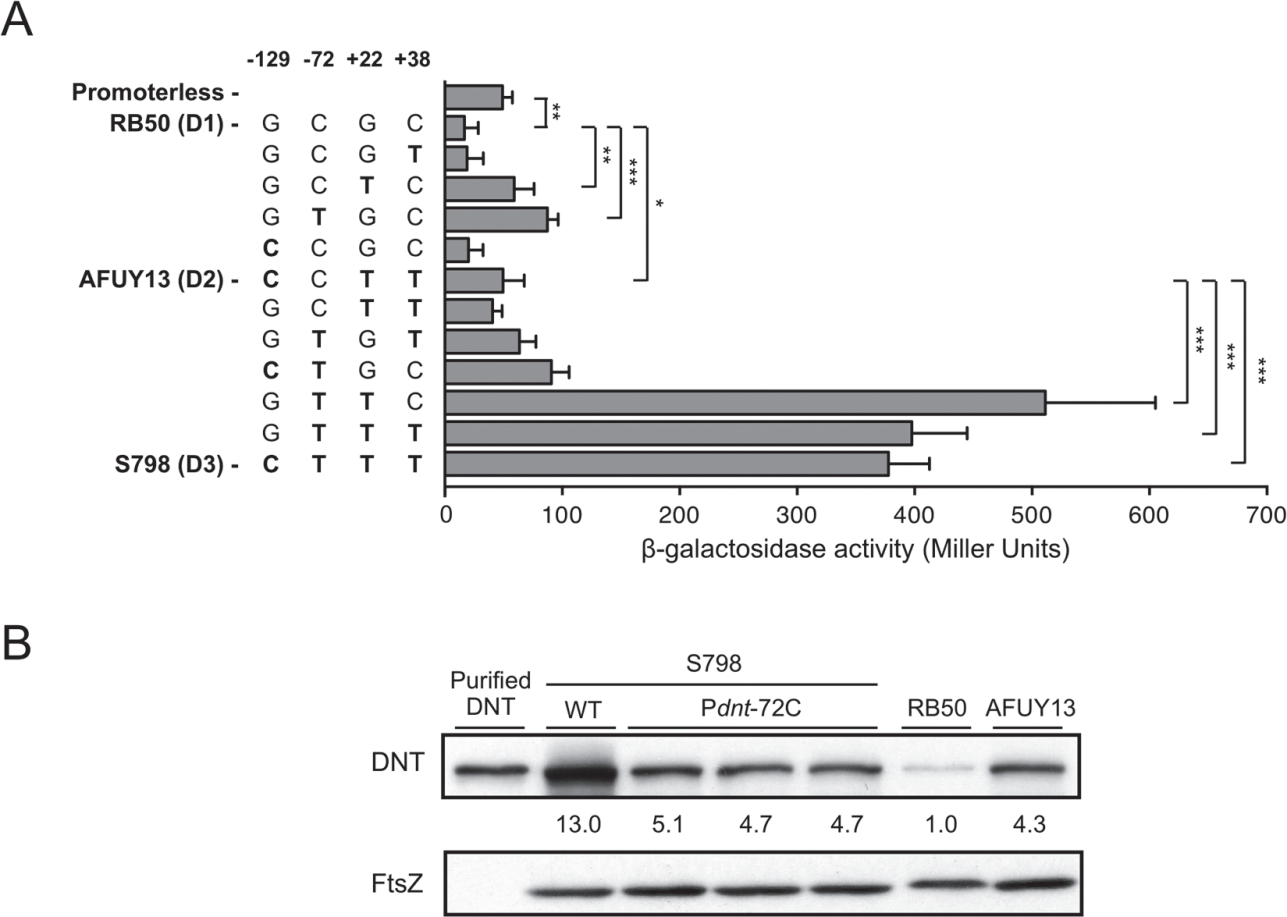
Activities of *dnt* promoters, in which nucleotides are substituted at positions -129, -72, +22, and/or +38. (A) Transcriptional activities of the promoter regions of *dnt* with or without nucleotide substitutions as indicated in the left column were estimated by the β-galactosidase reporter assay. The nucleotide substitutions were made on the basis of three typical promoter sequences, D1, D2, and D3, which are exemplified by RB50, AFUY13, and S798 strains, respectively. *, P < 0.01; **, P < 0.001; ***, P < 0.0001, compared between indicated samples. Each bar represents the mean + standard deviation of six samples. (B) Detection of DNT in whole-cell lysates by immunoblotting. RB50 (D1 type), AFUY13 (D2), S798 (D3), and an S798 derivative S798P*dnt*-72C having a nucleotide substitution of C for T at position -72 were cultivated for 12 h and whole-cell lysates were prepared and subjected to SDS-PAGE, followed by immunoblotting, as described in Materials and Methods. The amount of DNT in the samples was estimated as mentioned in the legend for Fig. 1B. Purified DNT was subjected to SDS-PAGE at 50 ng/lane to indicate the position of the toxin.

The results shown in Fig. 1 indicate that the SNPs at positions -129, -72, +22, and +38 in the region upstream of *dnt* influence the transcriptional level of the gene. To determine which base changes are crucial, we carried out the β-galactosidase reporter assay by using the reporter plasmid including the *dnt* promoter regions that harbor nucleotide substitutions at the positions in question, and examined their promoter activities (Fig. 2A). In these assays, the promoters with D2- and D3-type sequences showed 3.0 times and 22.6 times higher activity than the D1 type, respectively. The promoters having C at -72 and G at +22 were less active than a reporter plasmid without a promoter, suggesting that transcription is repressed in these promoters. The substitution between G and C at -129 did not affect the transcription activity (compare the results of the sequences “G C G C (D1 type)” and “C C G C”, “C C T T (D2 type)” and “G C T T” or “G T T T” and “C T T T”). Similarly, change from C to T at +38 did not enhance the promoter activity (see the results of the sequences “G C G C (D1 type)” and “G C G T”, “G C T C” and “G C T T”, “G T G C” and “G T G T”, or “G T T C” and “G T T T”). In contrast, substitutions of T for C at -72 and/or for G at +22 resulted in an increase of the transcription level of the reporter. The promoters carrying T at both -72 and +22 positions showed the same level of the promoter activity as that of D3 type. These results imply that the base changes from C to T at -72 and from G to T at +22 are crucial for high-level expression of *dnt*. Notably, when comparing the sequences of D2 and D3 types, substitution of T for C at -72 appeared to result in a marked increase of transcription activity, as judged by the β-galactosidase reporter assay. To confirm this, we generated a derivative of D3-type S798 strain (S798P*dnt*-72C) by substituting C for T at -72 in the region upstream of *dnt* on the chromosome (as a result, its SNP type becomes D2), and estimated the level of DNT production by immunoblotting (Fig. 2B). As expected, the amount of DNT produced by S798P*dnt*-72C was reduced to about 40% of that of wild-type S798, the level that corresponds to that of D2-type strain AFUY13. Quantitative RT-PCR also demonstrated that the *dnt* mRNA level of S798P*dnt*-72C was comparable to that of AFUY13 (Fig. 1C). These results imply that T at -72 is a key base to confer the highest transcriptional activity of *dnt* on D3-type strains like S798 and factors other than SNP at -72 are not involved in the difference in the level of DNT production between D2-type and D3-type bordetellae.

### DNT is not expressed by *B*. *bronchiseptica* strain isolated from mice

**Fig 3:**
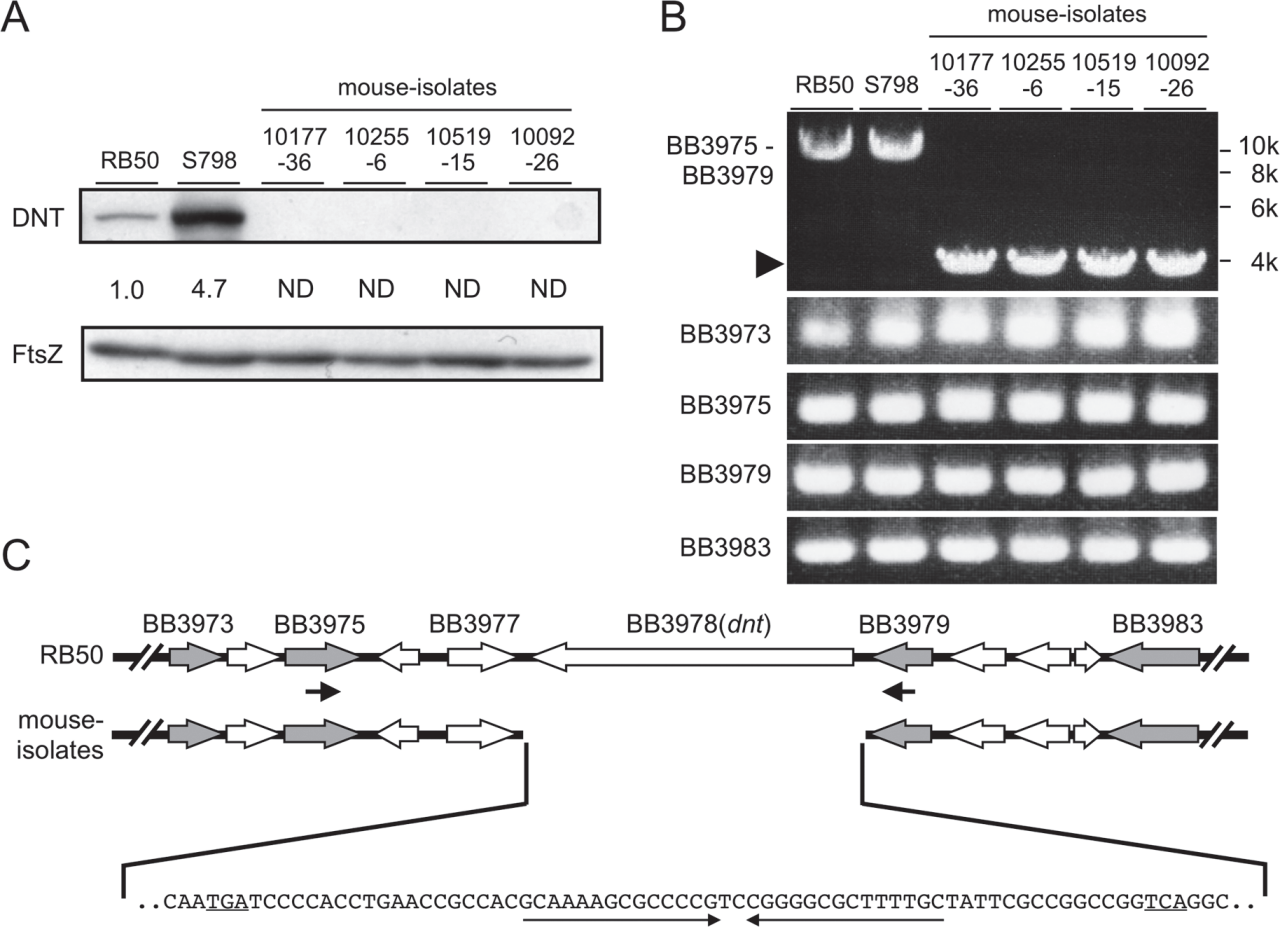
*dnt* is missing in mouse isolates. (A) Detection of DNT in whole-cell lysates of mouse isolates by immunoblotting. Whole-cell lysates were prepared and subjected to SDS-PAGE and immunoblotting as mentioned in the legend to Fig. 1B and Materials and Methods. The amount of DNT in the samples was estimated as mentioned in the legend to Fig. 1B. (B) PCR analysis of *dnt* and surrounding genes. Agarose gel electrophoresis of DNA fragments amplified by PCR targeting *dnt* and the surrounding genes. The genetic locus and genes amplified are indicated to the left. The PCR for the locus BB3975-BB3979 was carried out with primers corresponding to the locations indicated by thick black arrows in (C), which generates approximately ~9-kbp fragments from strains RB50 (a rabbit isolate) and S798 (a pig isolate). Note that the amplified fragments of ~4-kbp were obtained when mouse isolates were used as templates. (C) Schematic representation of the region surrounding *dnt*. White and shaded arrows indicate the open-reading frames with their relative lengths and translational directions. The shaded arrows are genes amplified by PCR shown in (B). The thick black arrows indicate the positions of primers used in PCR for amplification of the BB3975–3979 locus shown in (B). In mouse isolates, *dnt* is replaced by the indicated sequence. The translational stop codons of BB3977 and BB3979 are underlined. Thin arrows indicate the inverted repeat.

In the course of study to compare the ability to produce DNT among *B*. *bronchiseptica* strains, we found that mouse isolates produced no DNT (Fig. 3A). PCR to amplify a locus including *dnt* revealed that the amplified fragments from the mouse isolates were about 5 kbp shorter than those from RB50 and S798, rabbit and pig isolates, respectively, whereas genes located in the vicinity of *dnt*, BB3973, BB3975, BB3979, and BB3983, were amplified at the same sizes among all the strains (Fig. 3B). DNA sequencing of the short fragment amplified by PCR demonstrated that the mouse isolates lacked a region from 200 bp upstream to 93 bp downstream of *dnt*, and instead, a 63-bp fragment including a GC-rich palindrome of 14 bp was inserted (Fig. 3C).

### Distribution of promoter types of *dnt* in *Bordetella* spp.

Of 13 pig isolates of *B*. *bronchiseptica* that were examined in this study, nine carried a D2-type promoter region, three carried D3, and only one carried D1 (Fig. 1A and Table 1), implying that most *B*. *bronchiseptica* strains isolated from pigs may carry a D2 or D3 type. It is widely accepted that DNT is involved in the pathogenesis of *B*. *bronchiseptica* infection in pigs [22]. Therefore, there could be a correlation between the abilities of the bacteria to infect pigs and to produce a larger amount of DNT. To verify this, we examined the sequences upstream of *dnt* of strains that were deposited in the Genome database of the National Center for Biotechnology Information (NCBI, http://www.ncbi.nlm.nih.gov) (Table 1). Of 60 strains found in the database besides RB50 and S798, three were pig isolates, all of which carried a D2-type promoter. D2-type promoters were also found in three other strains that were isolated from monkey, guinea pig, and dog. D1 type was found in 27 strains. Eleven strains showed sequences distinct from D1, D2, and D3 types, but could be divided into three groups, which were tentatively designated as undefined type 1 (UT1) for seven strains, UT2 for three, and UT3 for one (S2 Fig.). For the remaining 16 strains, no *dnt* but sequences including the GC-rich palindrome reported in this study were found in the database. We next extended examples to *B*. *pertussis* and *B*. *parapertussis*, the sequences of which were obtained from the same database (S4 Table). All of these strains but one that was designated as UT4 were found to have a D1-type promoter. No strains harboring a D3-type promoter were found in the database of the three *Bordetella* species.

**Table 1:** SNP type of the *dnt* promoter in *Bordetella bronchiseptica*.

Strain	Origin	Geographic location	SNP^*a*^	ST^*d*^	Accession ID^*f*^
**RB50**	Rabbit	USA	D1	12	NC_002927.3
**S798**	Pig	Japan	D3	6	AP014582.1(This study)
**AFUY13**	Pig	Japan	D2	7	This study
**KG19**	Pig	Japan	D2	7	This study
**KHI11**	Pig	Japan	D2	7	This study
**KII13**	Pig	Japan	D3	6	This study
**KII24**	Pig	Japan	D3	6	This study
**MH110**	Pig	Japan	D2	7	This study
**R033**	Pig	Japan	D2	7	This study
**S807**	Pig	Japan	D1	ND^*e*^	This study
**STY136**	Pig	Japan	D2	7	This study
**STY137**	Pig	Japan	D2	7	This study
**TYA12**	Pig	Japan	D2	7	This study
**TYA13**	Pig	Japan	D2	7	This study
**10177–36**	Mouse	Japan	-^*b*^	8	This study
**10255–6**	Mouse	Japan	-	8	This study
**10519–15**	Mouse	Japan	-	8	This study
**10092–26**	Mouse	Japan	-	8	This study
1289	Monkey	South America	D2	32	NZ_HE983626.1
253	Dog	USA	D1	27	NC_019382.1
MO149	Human	USA	UT1^*c*^	15	NC_018829.1
D445	Human	USA	-	17	NZ_HE983627.1
Bbr77	Human	Germany	-	18	NZ_HE983628.1
345	Human	USA	-	21	JGWJ00000000.1
00-P-2730	Human	USA	UT1	34	JGWG00000000.1
00-P-2796	Human	USA	D1	27	JGWH00000000.1
3E44	Rabbit	USA	D1	10	JGWK00000000.1
7E71	Horse	USA	-	13	JGWL00000000.1
980	Unknown	Unknown	D1	10	JGWM00000000.1
99-R-0433	Human	USA	-	60	JGWN00000000.1
A1–7	Unknown	Unknown	D1	38	JGWO00000000.1
B18–5 (C3)	Unknown	Unknown	D1	14	JGWP00000000.1
C4	Rabbit	USA	D1	38	JGWQ00000000.1
CA90 BB02	Turkey	USA	-	8	JHBU00000000.1
CA90 BB1334	Turkey	USA	-	28	JGWR00000000.1
CARE970018BB	Pig	USA	D2	7	JGWS00000000.1
D756	Unknown	Unknown	D1	12	JGWT00000000.1
D989	Human	Unknown	D1	12	JGWU00000000.1
D993	Human	Unknown	D1	31	JGWV00000000.1
E010	Human	Unknown	D1	31	JGWW00000000.1
E012	Human	Unknown	D1	27	JGWX00000000.1
E013	Human	Unknown	D1	27	JGWY00000000.1
E014	Human	Unknown	-	ND	JGWZ00000000.1
F-1	Turkey	USA	UT1	65	JGXA00000000.1
F2	Turkey	USA	UT1	65	JGXB00000000.1
F4563	Human	USA	-	8	JGXC00000000.1
GA96–01	Human	USA	-	21	JGXD00000000.1
KM22	Pig	Hungary	D2	7	JNHR00000000.1
M435/02/3	Seal	Scotland	UT2^*b*^	11	JGXE00000000.1
M85/00/2	Seal	Caspian Sea	D1	33	JGXF00000000.1
MBORD591	Dog	USA	-	22	JGXG00000000.1
MBORD595	Dog	USA	D1	33	JGXH00000000.1
MBORD624	Horse	USA	D1	23	JGXI00000000.1
MBORD632	Horse	USA	D1	33	JGXJ00000000.1
MBORD635	Cat	USA	D1	62	JGXK00000000.1
MBORD665	Guinea Pig	USA	UT2	7	JGXL00000000.1
MBORD668	Guinea Pig	USA	UT2	7	JGXM00000000.1
MBORD670	Guinea Pig	USA	D2	7	JGXN00000000.1
MBORD675	Human	Germany	-	18	JGXO00000000.1
MBORD678	Guinea Pig	Australia	UT3^*c*^	14	JHBQ00000000.1
MBORD681	Koala	Australia	D1	ND	JGXP00000000.1
MBORD698	Koala	Australia	D1	ND	JGXQ00000000.1
MBORD707	Turkey	USA	UT1	29	JGXR00000000.1
MBORD731	Horse	Denmark	D1	6	JGXS00000000.1
MBORD762	Guinea Pig	Ireland	D1	14	JHBR00000000.1
MBORD782	Cat	Netherlands	D1	5	JGXT00000000.1
MBORD785	Dog	Netherlands	D2	7	JGXU00000000.1
MBORD839	Dog	Switzerland	D1	31	JGXV00000000.1
MBORD849	Pig	Netherlands	D2	7	JGXW00000000.1
MBORD901	Turkey	Germany	-	64	JGXX00000000.1
MO211	Human	USA	-	17	CAKT00000000.1
MO275	Human	USA	UT1	3	JHBS00000000.1
OSU054	Human	USA	-	28	JHBZ00000000.1
OSU095	Turkey	USA	-	28	JGXY00000000.1
OSU553	Turkey	USA	D1	35	JGXZ00000000.1
RB630	Rabbit	Hungary	D1	12	JGYA00000000.1
SBL-F6116	Human	USA	UT1	9	JHBT00000000.1
SO10328	Sea otter	USA	D1	23	JGYB00000000.1

^*a*^ SNP types of the region upstream of *dnt*.

^*b*^ no *dnt* in genomic DNA or shotgun DNA sequences.

^*c*^ undefined sequence types as shown in S2 Fig.

^*d*^ sequence types according to the *Bordetella* MLST database (http://pubmlst.org/bordetella).

^*e*^ not designated.

^*f*^ Four complete genome sequences and 58 whole-genome shotgun sequences were obtained from the NCBI website (http://www.ncbi.nlm.nih.gov/nuccore).

## Discussion

DNT is known as the major virulence factor of *B*. *bronchiseptica* causing pig atrophic rhinitis [23]. The toxin deamidates or polyaminates, and activates the members of the small GTPase Rho family, which results in the inhibition of osteoblastic differentiation, leading to atrophy of turbinate bone, the characteristic pathological lesion of atrophic rhinitis [7,8,23–26]. While it is well accepted that DNT is involved in the establishment of pig disease, the role of the toxin in diseases of mammals other than pigs is unknown.

In this study, we demonstrated that pig isolates of *B*. *bronchiseptica* produced more DNT than *B*. *pertussis* Tohama and *B*. *bronchiseptica* RB50, both of which are widely used as typical reference strains. Quantitative RT-PCR and β-galactosidase reporter assays revealed that the difference in DNT production reflected the difference in its transcription. Furthermore, we found the characteristic four SNPs at the promoter region of *dnt* and categorized them into three types, D1, D2, and D3, of which the ability to produce DNT increases in this order. Of the four SNPs, the two at positions -72 and +22 from the transcriptional start site were found to make synergistic and marked effects on the activity of *dnt* transcription (Fig. 2A): The transcriptional activities were highest and moderate when T was placed at both positions, like in D3 type, and at either position like in D2 type, respectively.

**Fig 4:**
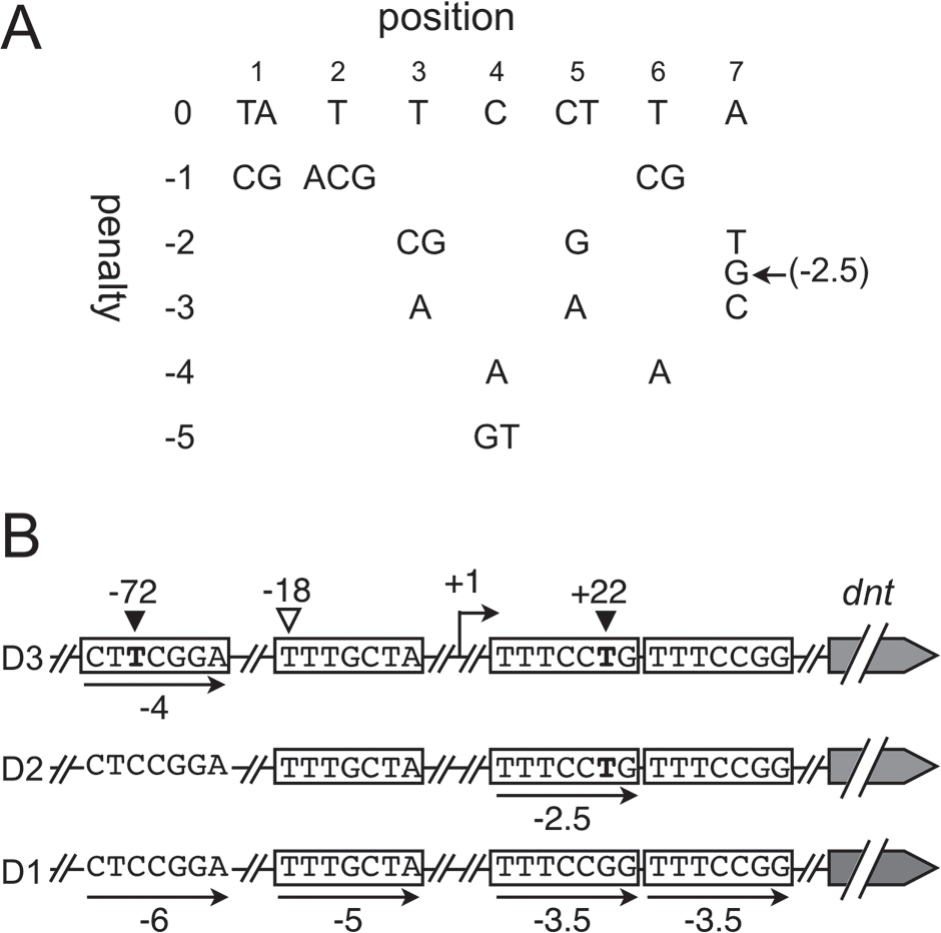
The putative BvgA-recognition sites upstream of *dnt*. (A) Table to evaluate the fitness of heptad sequences for BvgA-recognition site according to the algorithm of Merkel *et al*. [33]. The first row shows the heptad sequences of typical high-affinity binding sites for BvgA. Nucleotide substitutions shown in the table result in decreases of scores by the penalty values indicated in the first column. (B) Heptad sequences presumed as BvgA-recognition sites upstream of *dnt*. Arrows with numbers indicate the heptad sequences and scores calculated by the algorithm.

The transcription of *dnt* is known to be regulated by the BvgAS two-component system, in which BvgS phosphorylates the downstream transcriptional activator BvgA in response to environmental signals. Phosphorylated BvgA in turn binds to the promoter regions and activates transcription of virulence genes known as *vag*s (*vir*-activated genes) that, for example, encode filamentous hemagglutinin (*fhaB*), adenylate cyclase toxin (*cyaA*), pertussis toxin (*ptx*), and BvgAS *per se* (*bvgAS*), in addition to *dnt*. Many attempts to examine the nature of the BvgA-recognition sites upstream of *vags* have proposed consensus sequences [27–30]. Notably, inverted heptameric sequences composed of TTTCCTA, which are found in the promoter region of *fhaB*, are widely accepted as typical high-affinity binding sites for BvgA [30,31]. Merkel *et al*. created an algorithm to predict BvgA-binding sites by using the data of a mutational analysis of heptameric sequences in the *fhaB* promoter [32,33]. The algorithm enables us to estimate the fitness of 7-bp sequences as BvgA-recognition sites and to express it as a numerical score, where the *fhaB* binding site TTTCCTA has a score of 0, which is decreased by penalty values assigned to each base-substitution affecting BvgA-binding and/or the *fhaB* promoter activity (Fig. 4A). At the region upstream of *dnt*, there was a direct repeat predicted to be a BvgA-binding site, TTTCCTGTTTCCGG, at positions from +17 to +30, as reported previously [34]. One of the four SNPs that we found in this study is located in this direct repeat: D1 type of *dnt* promoter has G at position +22, whereas D2 and D3 types have T in place of G. In addition, we found two additional possible BvgA-binding sites at positions -18 to -12 and -74 to -68. D3 type possesses an SNP substituting T for C at position -72. Thus, in total, four BvgA-binding sites could be predicted upstream of *dnt* (Fig. 4B). The scores of possible BvgA-binding sites that were calculated according to the algorithm by Merkel *et al*. revealed that SNPs found in D2 and D3 at +22 and in D3 at -72 made the heptad sequences more suitable as BvgA-recognition sites, which is consistent with our results showing that the promoter activities become higher in the order of D1, D2, and D3. Although further analysis should be undertaken to delimit the actual BvgA-binding regions and examine the effects of the SNPs on *dnt* transcription activity, the scores of Merkel *et al*. suggest that the SNPs of D2 and D3 result in better matches to the BvgA-recognition site.

A previous study to analyze the genetic diversity of *B*. *pertussis*, *B*. *parapertussis*, and *B*. *bronchiseptica* by multilocus sequence typing (MLST) defined 32 sequence types (STs), which were categorized into four distinct lineages designated complexes I to IV [4]. *B*. *bronchiseptica* was divided into complexes I and IV, whereas *B*. *pertussis* and *B*. *parapertussis* human isolates were categorized as complexes II and III, respectively. *B*. *bronchiseptica* of complex IV mostly contained human isolates, and was more closely related to *B*. *pertussis* than those of complex I. *B*. *bronchiseptica* pig isolates examined for DNT production in this study were categorized as complex I, except S807 that was untyped by MLST. In addition, we found that all of the D3-type strains belong to ST6 and all of the D2 strains to ST7. This was the case even when strains on the database were examined (Table 1): all the pig isolates belong to D2 type and ST7. Although these results do not indicate that D2 and D3 types fully correspond to ST7 and ST6, respectively, because strains of ST7 but not D2 and ones of ST6 but not D3 were found in the database, the fact that D2- and D3-type strains all belong to ST7 and ST6, respectively, implies that the SNPs upstream of *dnt* are the result of evolutionary processes, not sporadic mutations. Similarly, all of the mouse isolates, which were found not to possess *dnt*, belong to ST8 of complex IV. This observation is consistent with previous studies that pointed out that *dnt* was missing or divergent in complex IV strains [3,4].

The distribution of the *dnt* promoter types in mammal isolates of bordetellae might reflect the importance of DNT in the pathogenesis of disease of each animal. *B*. *pertussis* and *B*. *parapertussis* human isolates mostly have D1-type promoter, which produces the least DNT, the role of which in whooping cough, a human disease of bordetellae, is unknown or limited, if any. In contrast, it is conceivable that almost all pig isolates belong to D2 and D3 types, which produce DNT at moderate and extreme levels, respectively, because the toxin causes turbinate bone atrophy, the characteristic lesion seen in atrophic rhinitis, pig infection by *B*. *bronchiseptica*. In addition, our results raise the possibility that DNT is not only responsible for causing the characteristic bone atrophy but also necessary for the bacteria to colonize the pig respiratory tract. This might be supported by a previous report showing that *dnt* mutants of *B*. *bronchiseptica* colonized the upper respiratory tract of pigs less than the parent strains [22].

It is known that *B*. *bronchiseptica*, *B*. *pertussis*, and *B*. *parapertussis*, despite sharing many virulence factors, vary in the pathogenic phenotypes including disease severity and host specificity. This diverse bacterial pathogenesis should have resulted from gene deletion and inactivation, as well as genomic translocation, during evolution of the subspecies. Our results shown in this study might indicate the fact that the difference in promoter activities of virulence genes caused by SNPs contributes, at least partly, to the determination of host specificity of *B*. *bronchiseptica*.

## Supporting Information

S1 Fig Schematic diagram of plasmid pT1T2-SD-*lacZ* for β-galactosidase reporter assay. Genes required for plasmid replication (*rep*), resistance to gentamicin (GmR) and plasmid mobilization (*mob*) are symbolized by solid arrows. β-galactosidase gene is symbolized by an open arrow. The promoter region of *dnt* is symbolized by an open box. The *rrnB* terminator region is indicated by a gray box.
(TIF)

S2 Fig Alignments of nucleotide substitutions upstream of *dnt*. Positions +1 and +47 indicate the start sites of transcription and translation, respectively. Positions -129, -72, +22, and +38 indicate the SNP positions among the D1, D2, and D3 types. BB and BP indicate *B*. *bronchiseptica* and *B*. *pertussis*, respectively. Four undefined types (UT) were found in the strains, whose sequences were deposited in the Genome database of NCBI.
(TIF)

S1 Table Bacterial strains used in this study. (DOC)

S2 Table Plasmids used in this study. (DOC)

S3 Table Primers used in this study. (DOC)

S4 Table Distribution of sequence type upstream of *dnt* in *Bordetella* spp. (DOC)
