# Supplementary figures and images for: Polymorphisms Influencing Expression of Dermonecrotic Toxin in Bordetella bronchiseptica
Source: PLoS One. 2015 Feb 2;10(2):e0116604. doi: 10.1371/journal.pone.0116604 (PMC4314077; doi:10.1371/journal.pone.0116604)

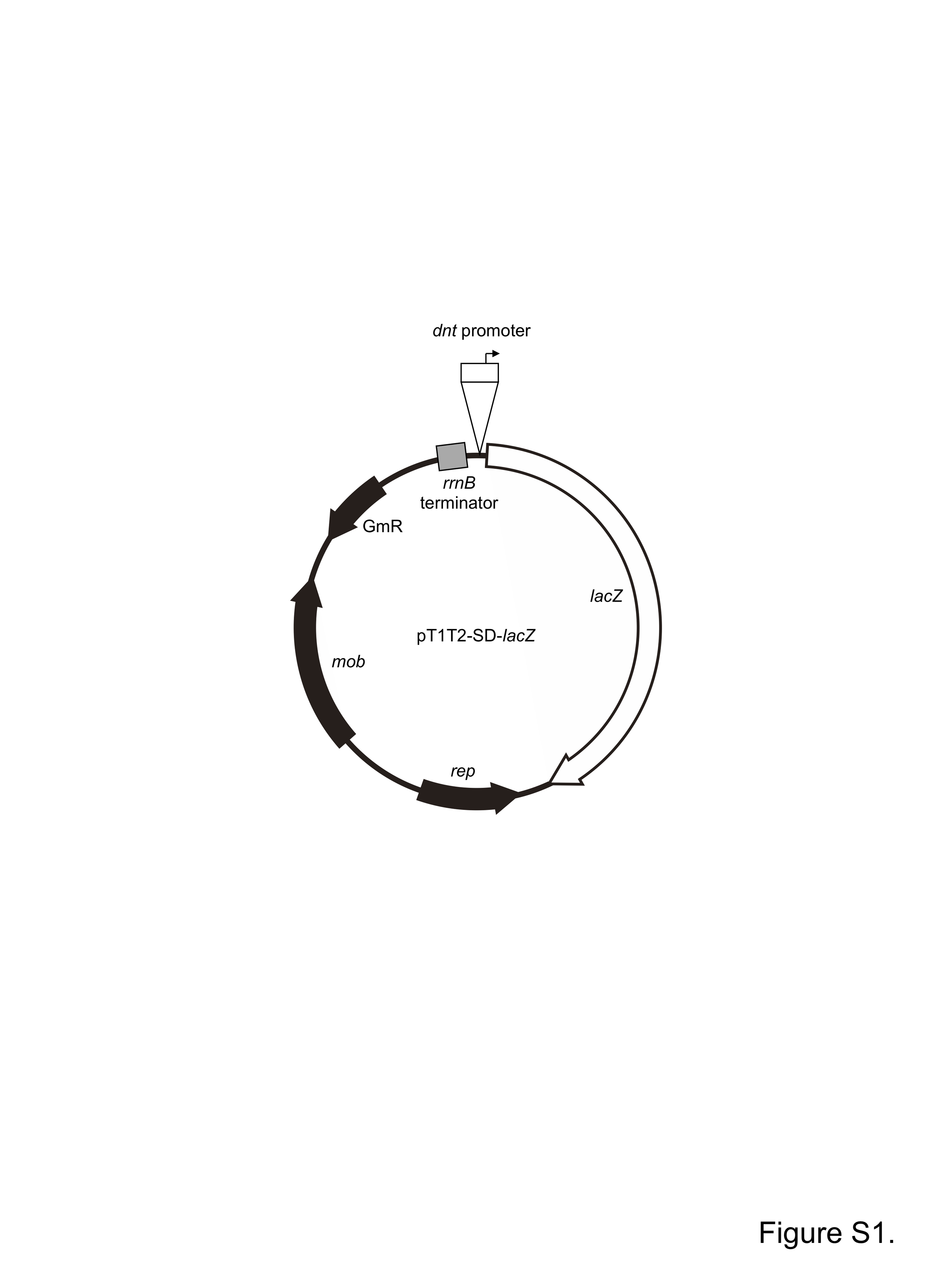

Supplement: S1 Fig — Genes required for plasmid replication (rep), resistance to gentamicin (GmR) and plasmid mobilization (mob) are symbolized by solid arrows. β-galactosidase gene is symbolized by an open arrow. The promoter region of dnt is symbolized by an open box. The rrnB terminator region is indicated by a gray box. (TIF) [file pone.0116604.s001.tif]

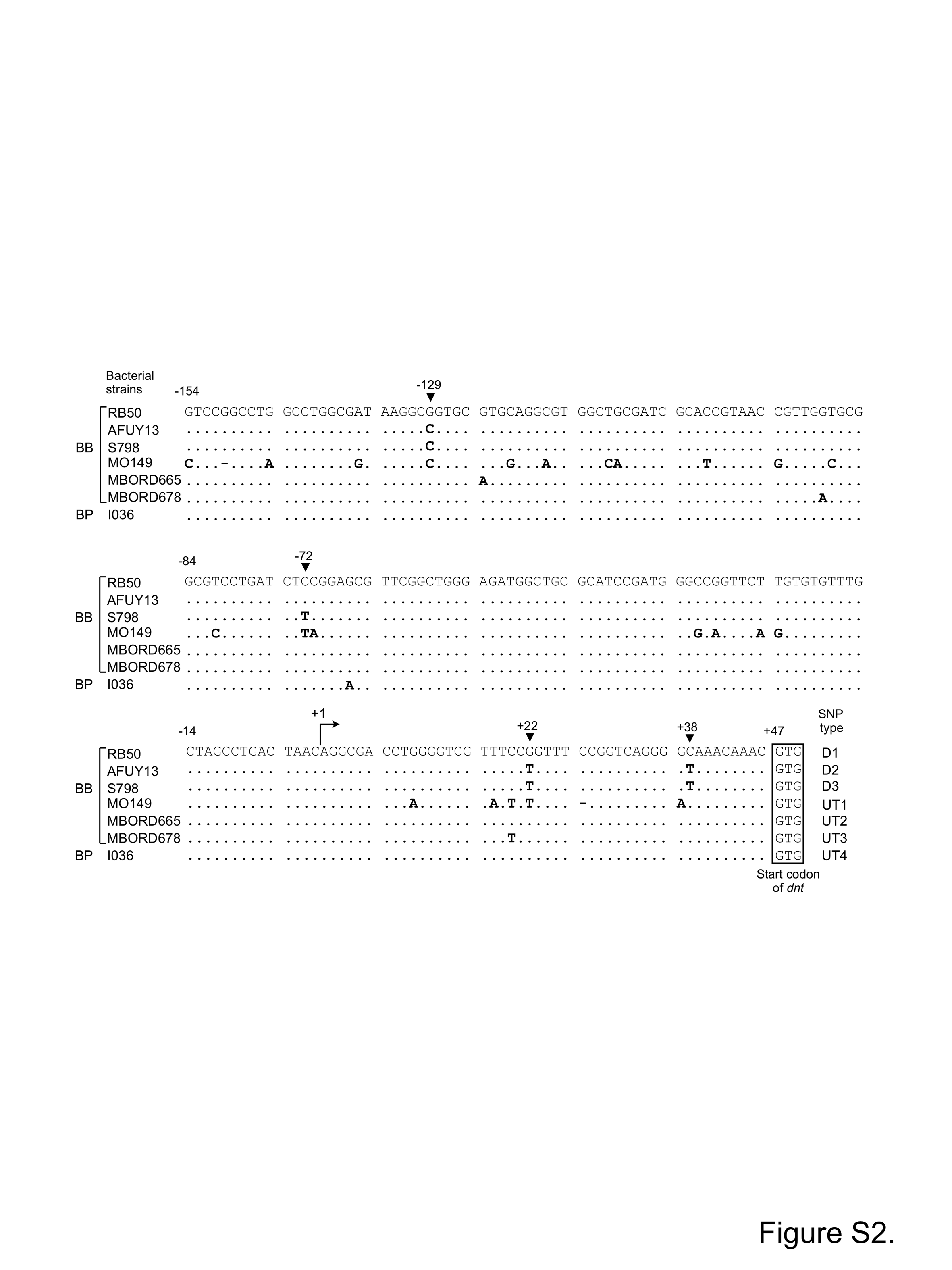

Supplement: S2 Fig — Positions +1 and +47 indicate the start sites of transcription and translation, respectively. Positions -129, -72, +22, and +38 indicate the SNP positions among the D1, D2, and D3 types. BB and BP indicate B. bronchiseptica and B. pertussis, respectively. Four undefined types (UT) were found in the strains, whose sequences were deposited in the Genome database of NCBI. (TIF) [file pone.0116604.s002.tif]
