# Supplementary material for: Polymorphisms Influencing Expression of Dermonecrotic Toxin in Bordetella bronchiseptica
Source: PLoS One. 2015 Feb 2;10(2):e0116604. doi: 10.1371/journal.pone.0116604 (PMC4314077; doi:10.1371/journal.pone.0116604)
Supplement: S1 Table — (DOC) [file pone.0116604.s003.doc]

| **TABLE S1.** Bacterial strains used in this study. | | | | |
| --- | --- | --- | --- | --- |
| **Strain** | | **Description** | **Origin** | **Source or reference** |
| *B. bronchiseptica* | | | | |
|  | S798 | Wild type, genome sequenced | Pig | Okada *et al*. (2014) |
|  | RB50 | Wild type, genome sequenced | Rabbit | Cotter *et al*. (1994) |
|  | S798P*dnt*-72C | S798 derivative containing the region upstream of *dnt* with -72C mutation |  | This study |
|  |  |  |  |  |
| *B. bronchiseptica* field strains isolated in Japan (isolation year) | | | | |
|  | AFUY13 | (1992) | Pig | This study |
|  | KG19 | (1993) | Pig | This study |
|  | KHI11 | (1997) | Pig | This study |
|  | KII13 | (1993) | Pig | This study |
|  | KII24 | (1993) | Pig | This study |
|  | MH110 | (1998) | Pig | This study |
|  | R033 | (1988) | Pig | This study |
|  | S807 | (1988) | Pig | This study |
|  | STY136 | (1998) | Pig | This study |
|  | STY137 | (1998) | Pig | This study |
|  | TYA12 | (1992) | Pig | This study |
|  | TYA13 | (1992) | Pig | This study |
|  | 10177-36 | (2010) | Mouse | This study |
|  | 10255-6 | (2010) | Mouse | This study |
|  | 10519-15 | (2010) | Mouse | This study |
|  | 10092-26 | (2010) | Mouse | This study |
|  |  |  |  |  |
| *B. pertussis* | |  |  |  |
|  | Tohama | Wild type, genome sequenced | Human | Parkhill *et al*. (2003) |
|  |  |  |  |  |
| *E. coli* | |  |  |  |
|  | DH5α | K-12 cloning strain |  | Laboratory collection |
|  | HB101 | K-12 cloning strain |  | Ehrmann *et al*. (1992) |
|  | W3110 | Wild-type strain used for amplification of *lacZ* |  | Hayashi *et al*. (2006) |
|  | DH5α λ*pir* | K-12 cloning strain of plasmid with R6K origin |  | Miller *et al*. (1988) |

1. Okada K, Ogura Y, Hayashi T, Abe A, Kuwae A, et al. (2014) Complete Genome Sequence of *Bordetella bronchiseptica* S798, an Isolate from a Pig with Atrophic Rhinitis. Genome Announc 2. doi:10.1128/genomeA.00436-14.

2. Cotter PA, Miller JF (1994) BvgAS-mediated signal transduction: analysis of phase-locked regulatory mutants of *Bordetella bronchiseptica* in a rabbit model. Infect Immun 62: 3381–3390.

3. Parkhill J, Sebaihia M, Preston A, Murphy LD, Thomson N, et al. (2003) Comparative analysis of the genome sequences of *Bordetella pertussis*, *Bordetella parapertussis* and *Bordetella bronchiseptica*. Nat Genet 35: 32–40. doi:10.1038/ng1227.

4. Ehrmann IE, Weiss AA, Goodwin MS, Gray MC, Barry E, et al. (1992) Enzymatic activity of adenylate cyclase toxin from *Bordetella pertussis* is not required for hemolysis. FEBS Lett 304: 51–56.

5. Hayashi K, Morooka N, Yamamoto Y, Fujita K, Isono K, et al. (2006) Highly accurate genome sequences of *Escherichia coli* K-12 strains MG1655 and W3110. Mol Syst Biol 2: 2006.0007. doi:10.1038/msb4100049.

6. Miller VL, Mekalanos JJ (1988) A novel suicide vector and its use in construction of insertion mutations: osmoregulation of outer membrane proteins and virulence determinants in *Vibrio cholerae* requires *toxR*. J Bacteriol 170: 2575–2583.

7. Kovach ME, Elzer PH, Hill DS, Robertson GT, Farris MA, et al. (1995) Four new derivatives of the broad-host-range cloning vector pBBR1MCS, carrying different antibiotic-resistance cassettes. Gene 166: 175–176.

8. Sekiya K, Ohishi M, Ogino T, Tamano K, Sasakawa C, et al. (2001) Supermolecular structure of the enteropathogenic *Escherichia coli* type III secretion system and its direct interaction with the EspA-sheath-like structure. Proc Natl Acad Sci USA 98: 11638–11643. doi:10.1073/pnas.191378598.
