# Supplementary material for: Polymorphisms Influencing Expression of Dermonecrotic Toxin in Bordetella bronchiseptica
Source: PLoS One. 2015 Feb 2;10(2):e0116604. doi: 10.1371/journal.pone.0116604 (PMC4314077; doi:10.1371/journal.pone.0116604)
Supplement: S2 Table — (DOC) [file pone.0116604.s004.doc]

| **TABLE S2.** Plasmids used in this study. | | |
| --- | --- | --- |
| **Plasmid** | **Description** | **Source or reference** |
| pCR2.1-TOPO | Ap^r^, Km^r^, cloning vector | Invitrogen |
| pRK2013 | Km^r^, helper plasmid containing *tra* and *mob* | Ehrmann *et al*. (1992) |
| pKK232-8 | Cm^r^, cloning vector | Amersham |
| pBBR1MCS-5 | Gm^r^, broad-host-range cloning vector | Kovach *et al*. (1995) |
| pABB-CRS2-GmA2 | Gm^r^, R6K-derived suicide vector containing *rpsL* | Sekiya *et al*. (2001) |
| pABB-CRS2-P*dnt*-72C | pABB-CRS2-GmA2 derivative containing the region upstream of *dnt* with -72C mutation | This study |
| pBBr01-SD | pBBR1MCS-5 derivative containing BBr01 promoter, cloning site and SD sequence | This study |
| pBBr01-SD-*lacZ* | pBBr01-SD derivative containing *lacZ* | This study |
| pT1T2-BBr01-SD-*lacZ* | pBBr01-SD-*lacZ* derivative containing *rrnB* terminater | This study |
| pT1T2-SD-*lacZ* | pT1T2-BBr01-SD-*lacZ* derivative without BBr01 promoter | This study |
| pT1T2-RB50 | pT1T2-SD-*lacZ* derivative containing the region upstream of *dnt* in RB50 | This study |
| pT1T2-S798 | pT1T2-SD-*lacZ* derivative containing the region upstream of *dnt* in S798 | This study |
| pT1T2-AFUY13 | pT1T2-SD-*lacZ* derivative containing the region upstream of *dnt* in AFUY13 | This study |
| pT1T2-RB50-1 | pT1T2-RB50 derivative containing the region upstream of *dnt* with +38T mutation | This study |
| pT1T2-RB50-2 | pT1T2-RB50 derivative containing the region upstream of *dnt* with +22T mutation | This study |
| pT1T2-RB50-3 | pT1T2-RB50 derivative containing the region upstream of *dnt* with -72T mutation | This study |
| pT1T2-RB50-4 | pT1T2-RB50 derivative containing the region upstream of *dnt* with -129C mutation | This study |
| pT1T2-RB50-5 | pT1T2-RB50 derivative containing the region upstream of *dnt* with +38T and +22T mutation | This study |
| pT1T2-RB50-6 | pT1T2-RB50 derivative containing the region upstream of *dnt* with +38T and -72T mutation | This study |
| pT1T2-RB50-7 | pT1T2-RB50 derivative containing the region upstream of *dnt* with +22T and -72T mutation | This study |
| pT1T2-RB50-8 | pT1T2-RB50 derivative containing the region upstream of *dnt* with -72T and -129C mutation | This study |
| pT1T2-RB50-9 | pT1T2-RB50 derivative containing the region upstream of *dnt* with +38T, +22T and -72T mutation | This study |

1. Ehrmann IE, Weiss AA, Goodwin MS, Gray MC, Barry E, et al. (1992) Enzymatic activity of adenylate cyclase toxin from *Bordetella pertussis* is not required for hemolysis. FEBS Lett 304: 51–56.

2. Kovach ME, Elzer PH, Hill DS, Robertson GT, Farris MA, et al. (1995) Four new derivatives of the broad-host-range cloning vector pBBR1MCS, carrying different antibiotic-resistance cassettes. Gene 166: 175–176.

3. Sekiya K, Ohishi M, Ogino T, Tamano K, Sasakawa C, et al. (2001) Supermolecular structure of the enteropathogenic *Escherichia coli* type III secretion system and its direct interaction with the EspA-sheath-like structure. Proc Natl Acad Sci USA 98: 11638–11643. doi:10.1073/pnas.191378598.
