# Supplementary material for: Polymorphisms Influencing Expression of Dermonecrotic Toxin in Bordetella bronchiseptica
Source: PLoS One. 2015 Feb 2;10(2):e0116604. doi: 10.1371/journal.pone.0116604 (PMC4314077; doi:10.1371/journal.pone.0116604)
Supplement: S3 Table — (DOC) [file pone.0116604.s005.doc]

| **TABLE S3.** Primers used in this study. | |
| --- | --- |
| **Name** | **Sequence** |
| Adk-F | 5’-AGCCGCCTTTCTCACCCAACACT-3’ |
| Adk-R | 5’-TGGGCCCAGGACGAGTAGT-3’ |
| FumC-F | 5’-CGTGAACCGGGGCCAGTCGTC-3’ |
| FumC-R | 5’-GGCCAGCCAGCGCACATCGTT-3’ |
| GlyA-F | 5’-CAACCAGGGCGTGTACATGGC-3’ |
| GlyA-R | 5’-CCGCGATGACGTGCATCAG-3’ |
| TyrB-F | 5’-CGAGACCTACGCTTATTACGAT-3’ |
| TyrB-R | 5’-TGCCGGCCAGTTCATTTT-3’ |
| Icd-F | 5’-CTGGTCCACAAGGGCAACAT-3’ |
| Icd-R | 5’-ACACCTGGGTGGCGCCTTC-3’ |
| PepA-F | 5’-CGCCCCAGGTTGAAGAAAATCGTC-3’ |
| PepA-R | 5’-ATCAGGCCCACCACATCCAG-3’ |
| Pgm-F | 5’-CGCCCATGTCACCAGCACCGA-3’ |
| Pgm-R | 5’-CGCCGTCTATCGTAACCAG-3’ |
| S798-DNTr-Up-S | 5’-atcgtctatatcgaccgggc-3’ |
| S798-DNTr-Up-S+ | *5’-gatccgagctctcccatcgtctatatcgaccgggc-3’ |
| S798-DNTr-Down2-AS | §5’-aacgctccggagatcagga-3’ |
| S798-DNTr-Up2-S | 5’-gcctgcaggacgaatggata-3’ |
| S798-DNTr-Down-AS | 5’-gcgccttgacatactcgaat-3’ |
| S798-DNTr-Down-AS+ | *5’-atttgtggaattcccgcgccttgacatactcgaat-3’ |
| *Pst*I-GmR-S | 5’-CTGCAGTGCCCATGGACGCACACC-3’ |
| *Pst*I-GmR-AS | 5’-CTGCAGTCCCCGAAAAGTGCCACCTG-3’ |
| RT-3978-F | 5’-gcgcgtggaatcgaaaatc-3’ |
| RT-3978-R | 5’-tcatggagctggaacatggtg-3’ |
| RT-*recA*-F | 5’-gccagggcaaggacaatgt-3’ |
| RT-*recA*-R | 5’-cgatggccatttccttgtg-3’ |
| DNT-5RACE-RT | 5’-ataccaggtcgctga-3’ |
| DNT-5RACE-S1 | 5’-tcgagcctgagtcattgatc-3’ |
| DNT-5RACE-A1 | 5’-gaaccatgtacgagcatcct-3’ |
| DNT-5RACE-S2 | 5’-agtggttgatcgcactcgat-3’ |
| DNT-5RACE-A2 | 5’-gaattcggccttctctcgt-3’ |
| BB3973-S | 5’-TTACATCATAACGCCGTTGG-3’ |
| BB3973-AS | 5’-CACAGCATCGAAACCTGGAT-3’ |
| BB3975-S | 5’-GGTATTGGGGGAGGAAATGT-3’ |
| BB3975-AS | 5’-CTTCGGCTTCGTGGAAGTAG-3’ |
| BB3979-S | 5’-CCGTATTCCGAACGCTACAG-3’ |
| BB3979-AS | 5’-GACCGACAGGTTGATCGTCT-3’ |
| BB3983-S | 5’-AGCAGGTCATCGTGTTTTCC-3’ |
| BB3983-AS | 5’-AGCAGATAGCGCTCTTCGTC-3’ |
| BB3979-AS_reverse | 5’-AGACGATCAACCTGTCGGTC-3’ |
| BB3979-S-2 | 5’-CTCAATGACCTGGTGGTAGG-3’ |
| *Xho*I-PBBr01-SD-S+ | $5’-CCAATACGCCTCGAGAGGCCGACTTGACAAGCTG-3’ |
| *Sma*I-PBBr01-SD-AS+ | $5’-CCCGGGGTCCTCCTCGACCGGTTCTGAGCCAGGATCAAACTC-3’ |
| *Xho*I-pBBR1MCS-AS+ | 5’-CTCGAGGCGTATTGGGCGCATGCAT-3’ |
| SD-*Sma*I-pBBR1MCS-S+ | 5’-GAGGAGGAGCCCGGGCGGTGAATCCGTTAGCGAGG-3’ |
| *Ecoli*-*lacZ*-S+ | #5’-gtcgaggaggaccccatgaccatgattacggattcac-3’ |
| *Ecoli*-*lacZ*-AS+ | #5’-acggattcaccgcccgatttccttacgcgaaatacgg-3’ |
| Promoter-T1-S | 5’-tcggcagaatgcttaatg-3’ |
| Promoter-T1-AS | 5’-catggaagccatcacaa-3’ |
| *rrnB*-T1-termi-S2+ | †5’-taagcattctgccgaatgcgagagtagggaact-3’ |
| *rrnB*-T2-termi-AS2+ | †5’-gtgatggcttccatgcagctgctttcctgatgc-3’ |
| SD-*lacZ*-S | 5’-accggtcgaggaggacc-3’ |
| SD-*lacZ*-AS | 5’-ctcgaggcgtattgggcg-3’ |
| DNT-P-SD-S+ | ¥5’-ccaatacgcctcgaggtccggcctggcctg-3’ |
| RB50-DNT-P-SD-AS+ | ¥5’-tcctcctcgaccggtgtttgtttgcccctgaccg-3’ |
| S798-DNT-P-SD-AS+ | ¥5’-tcctcctcgaccggtgtttgtttacccctgaccg-3’ |
| RB50-DNT-P-72T-S | §5’-gcggcgtcctgatcttcggagcgttcggctg-3’ |
| RB50-DNT-P-72T-AS | §5’-cagccgaacgctccgaagatcaggacgccgc-3’ |
| RB50-DNT-P-129C-S | §5’-gcctggcgataaggccgtgcgtgcaggcgtg-3’ |
| RB50-DNT-P-129C-AS | §5’-cacgcctgcacgcacggccttatcgccaggc-3’ |
| RB50-DNT-P+22T-S | §5’-ctggggtcgtttccgttttccggtcaggggc-3’ |
| RB50-DNT-P+22T-AS | §5’-gcccctgaccggaaaacggaaacgaccccag-3’ |
| RB50-DNT-P+38T-S | §5’-gtttccggtcaggggtaaacaaacaccggtc-3’ |
| RB50-DNT-P+38T-AS | §5’-gaccggtgtttgtttacccctgaccggaaac-3’ |

* Underlined regions represent sequence overlapping each terminal region of the pABB-CRS2-GmA2 digested with *Sma*I.

§ Underlined bases represent nucleotides specifying the point mutation.

$ Underlined regions represent sequence overlapping each terminal region of a DNA fragment that was amplified by inverse PCR with pBBR1MCS-5 as a template and the combination of primers *Xho*I-pBBR1MCS-AS+ and SD-*Sma*I-pBBR1MCS-S+.

# Underlined regions represent sequence overlapping each terminal region of pBBr01-SD digested with *Sma*I.

† Underlined regions represent sequence overlapping each terminal region of a DNA fragment that was amplified by inverse PCR with pBBr01-SD-*lacZ* as a template and the combination of primers Promoter-T1-S and Promoter-T1-AS.

¥ Underlined regions represent sequence overlapping each terminal region of linearized pT1T2-BBr01-SD-*lacZ* without BBr01 promoter.
