# Supplementary material for: Polymorphisms Influencing Expression of Dermonecrotic Toxin in Bordetella bronchiseptica
Source: PLoS One. 2015 Feb 2;10(2):e0116604. doi: 10.1371/journal.pone.0116604 (PMC4314077; doi:10.1371/journal.pone.0116604)
Supplement: S4 Table — (DOC) [file pone.0116604.s006.doc]

| **TABLE S4.** Distribution of sequence type upstream of *dnt* in *Bordetella* spp. | | | | | | |
| --- | --- | --- | --- | --- | --- | --- |
| **Species** | **Strain** | **Origin** | **Geographic location** | **SNP***^a^* | **ST** | **Accession ID***^c^* |
| *B. parapertussis* | 12822 | Human | Germany | D1 | 19 | NC_002928.3 |
| *B. parapertussis* | Bpp5 | Sheep | New Zealand | D1 | 16 | NC_018828.1 |
| *B. pertussis* | Tohama I | Human | Japan | D1 | 1 | BX470248.1 |
| *B. pertussis* | CS | Human | China | D1 | 1 | NC_017223.1 |
| *B. pertussis* | 18323 | Human | USA | D1 | 24 | NC_018518.1 |
| *B. pertussis* | B0558 | Human | Netherlands | D1 | 2 | ADKR00000000.1 |
| *B. pertussis* | B1193 | Human | Netherlands | D1 | 1 | ADKS00000000.1 |
| *B. pertussis* | B1831 | Human | Netherlands | D1 | 2 | ADKT00000000.1 |
| *B. pertussis* | B1834 | Human | Netherlands | D1 | 2 | ADKU00000000.1 |
| *B. pertussis* | B1917 | Human | Netherlands | D1 | 2 | ADKV00000000.1 |
| *B. pertussis* | B1920 | Human | Netherlands | D1 | 2 | ADKW00000000.1 |
| *B. pertussis* | 3356847 | Unknown | Unknown | D1 | 2 | JGWI00000000.1 |
| *B. pertussis* | H934 | Human | Unknown | D1 | 2 | JGWF00000000.1 |
| *B. pertussis* | B200 | Human | USA | D1 | 1 | JGWE00000000.1 |
| *B. pertussis* | STO1-SEAT-0004 | Human | USA | D1 | 2 | AXSJ00000000.2 |
| *B. pertussis* | STO1-CHOM-0012 | Human | USA | D1 | 2 | AXRU00000000.2 |
| *B. pertussis* | STO1-CNMC-0004 | Human | USA | D1 | 2 | AXSV00000000.2 |
| *B. pertussis* | STO1-CHOC-0021 | Human | USA | D1 | 2 | AXRW00000000.2 |
| *B. pertussis* | STO1-CHOC-0019 | Human | USA | D1 | 2 | AXRX00000000.2 |
| *B. pertussis* | STO1-CHOC-0018 | Human | USA | D1 | 2 | AXRY00000000.2 |
| *B. pertussis* | STO1-CHOC-0017 | Human | USA | D1 | 2 | AXRZ00000000.2 |
| *B. pertussis* | STO1-CHOC-0016 | Human | USA | D1 | 2 | AXSA00000000.2 |
| *B. pertussis* | STO1-CHOC-0008 | Human | USA | D1 | 2 | AXRV00000000.2 |
| *B. pertussis* | STO1-CHLA-0011 | Human | USA | D1 | 2 | AXSP00000000.2 |
| *B. pertussis* | STO1-CHLA-0006 | Human | USA | D1 | 2 | AXSF00000000.2 |
| *B. pertussis* | I176 | Human | USA | D1 | 2 | AXSG00000000.2 |
| *B. pertussis* | I036 | Human | USA | UT4*^b^* | 2 | AXSH00000000.2 |
| *B. pertussis* | I002 | Human | USA | D1 | 2 | AXSI00000000.2 |
| *B. pertussis* | H973 | Human | USA | D1 | 2 | AXSK00000000.2 |
| *B. pertussis* | H921 | Human | USA | D1 | 2 | AXSM00000000.2 |
| *B. pertussis* | H939 | Human | USA | D1 | 2 | AXSL00000000.2 |
| *B. pertussis* | CHLA-26 | Human | USA | D1 | 2 | AXSB00000000.2 |
| *B. pertussis* | CHLA-20 | Human | USA | D1 | 2 | AXSC00000000.2 |
| *B. pertussis* | CHLA-15 | Human | USA | D1 | 2 | AXSD00000000.2 |
| *B. pertussis* | CHLA-13 | Human | USA | D1 | 2 | AXSE00000000.2 |
| *B. pertussis* | STO1-SEAT-0007 | Human | USA | D1 | 2 | AXSQ00000000.2 |
| *B. pertussis* | STO1-SEAT-0006 | Human | USA | D1 | 2 | AXSR00000000.2 |
| *B. pertussis* | 2371640 | Human | USA | D1 | 2 | AXSS00000000.2 |
| *B. pertussis* | 2356847 | Human | USA | D1 | 2 | AXST00000000.2 |
| *B. pertussis* | 2250905 | Human | USA | D1 | 2 | AXSU00000000.2 |
| *B. pertussis* | CHLA-11 | Human | USA | D1 | 2 | AYXH00000000.1 |

*^a^* SNP types of the region upstream of *dnt.*

*^b^* undefined sequence types as shown in Figure S2.

*^c^* Five complete genome sequences and 36 whole-genome shotgun sequences were obtained from the NCBI website (http://www.ncbi.nlm.nih.gov/nuccore).
